# The Italian landscape of digital therapeutics in a European context

**DOI:** 10.3389/fdgth.2026.1749302

**Published:** 2026-06-11

**Authors:** Giulia Ivagnes, Michela Di Pietro, Valentina Lacalamita, Davide De Pascale, Andrea Oliva

**Affiliations:** 1 Viatris Italia, Product Safety & Risk Management (PSRM), Milan, Italy; 2 Viatris Italia, Regulatory Affairs, Milan, Italy; 3 Viatris Italia, Medical Affairs, Milan, Italy; 4 Viatris Italia, Quality Assurance, Milan, Italy

**Keywords:** clinical trials, digital health, DTx (digital therapeutics), European Union, Italy, medical device regulation (UE) 2017/745, patient safety

## Abstract

**Introduction and aim:**

Digital Therapeutics (DTx) are emerging as a key component of the modern healthcare landscape, offering evidence-based therapeutic interventions powered by software. Unlike wellness apps, DTx require robust clinical validation to demonstrate their efficacy in managing and treating diseases such as diabetes, anxiety, depression, and hypertension. Clinical trials evaluating DTx follow rigorous methodologies similar to traditional drug trials, including randomized controlled trials (RCTs), but also present unique challenges, such as the need for software updates and decentralized patient management. The aim of this narrative review is to examine the European landscape of clinical trials and the associated regulatory framework for DTx, followed by a focus on the current situation in Italy.

**Methods and results:**

To contextualize the development of digital therapeutics, existing literature was first reviewed to identify key therapeutic areas. A targeted analysis of clinical trials performed in Italy was conducted using ClinicalTrials.gov as the data source. Predefined keywords were applied, and retrieved records were screened based on relevance to DTx and applicability to the Italian context. Studies were included if they investigated digital interventions with therapeutic intent and were conducted in Italy. Following screening, 13 DTx-related clinical trials were identified, including 11 interventional and 2 observational studies. All interventional studies were randomized controlled trials at different stages of development. The analysis revealed heterogeneity in study design regarding control arm strategies, with approaches ranging from standard of care and active controls to no-treatment comparators and digital placebo conditions. Regulatory frameworks for DTx have evolved significantly, with the European Medical Device Regulation (MDR 2017/745) now recognizing stand-alone software as a medical device. This shift emphasizes the importance of regulatory compliance, risk classification, and continuous monitoring to ensure patient safety and therapeutic effectiveness. We also examined post-market surveillance responsibilities to provide an overview of key opportunities and considerations for stakeholders interested in commercializing a digital therapeutic.

**Discussion and conclusion:**

As DTx continue to gain traction, their integration into healthcare systems will depend on clinical evidence, regulatory adaptation, and user engagement. Future developments will likely focus on optimizing trial methodologies and enhancing digital endpoints to improve patient outcomes.

## Introduction and aim

The aim of this narrative review is to assess the current EU landscape for digital therapeutics, with a specific focus on the Italian framework in comparison with other EU territories. This strategic review is intended to support stakeholders interested in commercializing a digital therapeutic in Italy, with particular attention to regulatory requirements and product management considerations. The need to focus specifically on the Italian context arises from the fact that the regulatory framework governing Digital Therapeutics (DTx) in Italy is currently less clearly defined and structured than in other European countries. Although the applicable regulations are established at the European level, the authority responsible for the evaluation, approval, market access, pricing, and reimbursement of DTx in Italy operates at the national level. This regulatory configuration may limit market entry and pose additional challenges for the stakeholders interested in commercializing a digital therapeutic in Italy.

**Figure 1:**
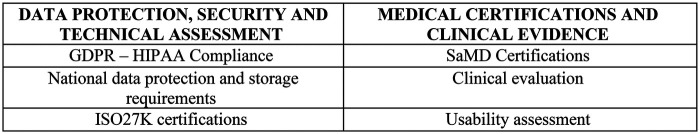
List of common requirements shared by EU countries.

DTx are evidence-based therapeutic interventions driven by high-quality software programs to prevent, manage, or treat a medical disorder or disease (1). Today, DTx is a recognized component of the scientific landscape, representing a novel and promising approach to treatment.

It should be noted that DTx are based on software programs and can be considered a kind of “software as a medical device”. DTx can be considered a subset of digital health, a broad category which includes a wide range of products used across the wellness and healthcare industry (2). DTx differ from common wellness apps or medication reminder tools in that they require robust clinical evidence to substantiate their intended use and demonstrate an impact on disease status.

The application of DTx is becoming increasingly prevalent, encompassing a wide range of physical and behavioural conditions, predominantly chronic. These include diabetes, anxiety disorder, depression, insomnia, attention-deficit/hyperactivity disorder (ADHD), substance use disorder, hypertension, chronic obstructive pulmonary disease and oncology treatment management (3).

For a digital therapeutic to be registered as a medical device and placed on the market, the manufacturer must be able to guarantee its safety and efficacy. Its use must therefore be safe for the patient, and it must also provide a statistically significant clinical benefit. In order to design a clinical study capable of meeting these requirements and producing meaningful results, manufacturers of digital therapeutics can rely on two key documents: the first is the ISO 14155 standard, entitled Clinical investigation of medical devices for human subjects – Good Clinical Practice; the second is the MDCG 2020-1 guideline Guidance on Clinical Evaluation (MDR)/Performance Evaluation (IVDR) of Medical Device Software issued in March 2020.

A key aspect for stakeholders interested in commercializing a digital therapeutic concerns the clinical trials supporting market entry for new DTx products.

Clinical trials designed to evaluate DTx share many features with traditional drug trials, while they also raise some unique challenges associated with the digital and interactive nature of these interventions (4). DTx trials are most often designed as either pilot or pivotal studies, each aimed at assessing different aspects of the therapy to ensure its safety and efficacy (5).

Pilot studies are the first steps that determine technical performance, usability, and basic safety. They very often relate to small population with the idea to optimize the design of the DTx and resolve any technical or methodological issues which can impact future trial stages. Modifications in the therapeutic algorithm or user interface have been quite common in this phase to maximize the treatment for better adherence and overall effectiveness (5).

Pivotal studies involve larger patient population and are designed to capture comprehensive data on the safety and effectiveness of DTx, similar to Phase III drug trials. These studies are necessary for gaining regulatory approval; therefore, they require strict inclusion and exclusion criteria, with rich data collection of a primary and secondary endpoint or key findings (5).

Randomized controlled trials are the gold standard for both DTx and drug studies, ensuring unbiased results by comparing the experimental intervention against a control group. These studies can be open-label, single-blind, double-blind, or triple-blind, each progressively minimizing biases (6). A key innovation in the trials of DTx involves the use of digital endpoints, i.e., real-time metrics collected through connected devices offering continuous monitoring and detailed insight into patient health not afforded by traditional endpoints (7).

Unique to DTx trials is the fact that software updates may be required during a study: this may be necessary to update the device-patient interface, introduce new device features or improve the user experience. Such situations would need extensive planning to make sure that the updates would not affect the integrity of the data and additional regulatory reviews (5). The other distinctive characteristic of DTx trials involves decentralized settings, where the patients are treated remotely (6). The decentralized approach requires the patients to be highly involved in the treatment process, making user interaction with the software crucial for accurate data collection (8).

## Methods and results

To frame the current landscape of digital therapeutics, we first considered the broader evidence base available in the literature. In particular, previously published reviews (9) were used as a contextual starting point to identify the main therapeutic areas in which DTx are currently being developed and investigated.

Building on this general overview, we conducted a targeted analysis focusing on clinical trials involving DTx within the Italian context. For this purpose, ClinicalTrials.gov was used as the primary data source.

The search was conducted using a set of predefined keywords, including “Digital Therapies”, “Digital Therapy”, “Digital Therapeutics”, “Digital Health”, “Digital Intervention”, “mHealth”, “eHealth”, and “Wearable Devices”. Retrieved records were screened to identify studies specifically involving digital therapeutics.

The initial search yielded a broad set of records. These were subsequently screened to identify studies specifically involving digital therapeutics.

Studies were included if they:

1. investigated a digital intervention with a clear therapeutic intent;
2. were conducted in Italy;

Studies were excluded if they:

1. focused on general digital health tools without a defined therapeutic objective (e.g., wellness or lifestyle applications),
2. did not involve a clinical evaluation component, or
3. were not conducted within the Italian context.

Following this selection process, a final set of 13 studies was identified and included in the analysis, of which 11 were interventional and 2 were observational. All interventional studies were randomized controlled trials (RCTs). Among these, 3 studies had been completed, 3 were actively recruiting, 1 was active but not recruiting, and 4 had not yet started recruitment.

The results of this analysis are summarized in Table 1.

**Table 1:** Digital therapeutics clinical trials conducted in Italy.

Study Name	Trial ID	Study Status	Study Type	Sponsor	Allocation	Model	Masking
Virtual Therapeutics for MCI (VR-MCI)	NCT06079411	Active-recruiting	Interventional	Istituto Auxologico Italiano	Randomized	Interventional-parallel assignment	None (open label)
KidneYou - Innovative Digital Therapy	NCT05286632	Active-recruiting	Interventional	Advice Pharma Group srl	Randomized	Interventional-parallel assignment	None (open label)
Digital Therapy in Shoulder Rehabilitation	NCT05230056	Completed	Interventional		Randomized	Interventional-parallel assignment	None (open label)
Telerehabilitation in Progressive Multiple Sclerosis	NCT06485115	Not yet recruiting	Interventional	Università di Verona	Randomized	Interventional-parallel assignment	Single (outcomes assessor)
DEMETRA - ADVICE-002-2022	NCT05394779	Active-not recruiting	Interventional	Advice Pharma Group srl	Randomized	Interventional-parallel assignment	None (open label)
Assessment of Efficacy							
and Safety of Amicomed®, for the Management of Essential Hypertension	NCT06091176	Not yet recruiting	Interventional	Newel Health SRL	Randomized	Interventional-parallel assignment	Double (investigator, outcome assessor)
CARINAE for Stress Relief in Perioperative Care (CARINAE)	NCT05184725	Completed	Interventional	Adhera Health, Inc.	Randomized	Interventional-parallel assignment	None (open label)
Telepsychiatry for Social Isolation in Youths (SOLITAIRE)	NCT06138301	Active-recruiting	Interventional	Azienda Ospedaliera Universitaria Integrata Verona	Randomized	Interventional-parallel assignment	Single (outcomes assessor)
Sleep and Eating Behaviours in Adolescents (SANA)	NCT06472466	Not yet recruiting	Interventional	University of Padova	Randomized	Interventional-parallel assignment	Single (outcomes assessor)
Investigating Neurocognitive, Motor and Biological Effects of MindLenses Professional in Neurological Diseases (MindLensesN)	NCT05826626	Completed	Interventional	IRCCS San Camillo, Venezia, Italy	Randomized	Interventional-parallel assignment	Single (participant)
Validation of a Digital Intervention to Rehabilitate Cognitive Resources (MI-RICORDO)	NCT07064226	Not yet recruiting	Interventional	IRCCS Fondazione Don Carlo Gnocchi ONLUS; Center of Advance Diagnostic and Therapy (CADiTeR)	Randomized	Interventional-parallel assignment	Single (outcomes assessor)

An examination of the study designs adopted in the identified trials highlights a heterogeneous range of approaches, particularly with regard to the definition and implementation of control arm strategies.

In the context of a randomized controlled clinical trial for digital therapies, the control arm represents a particularly delicate aspect. In effect, randomized controlled trials (RCTs) necessitate the comparison of drug therapy candidates with a control group. The specific nature and type of the control group remain a topic of ongoing debate. During the designing phase of the study, it is important to define the treatment of the control arm for an accurate assessment of the efficacy of the experimental therapy, as the latter must be demonstrated to be at least as effective as the treatment given to the control group to be validated (10).

In contrast to the methodology employed in numerous conventional clinical trials, which utilize a control group treated with the best available therapeutic alternative or a placebo, DTx may have different forms of comparators and control strategies:

### Standard of care (treatment as usual, TAU)

This involves the evidence-based established treatment for a given disease to be administered on the control population. This methodological approach enables the evaluation of whether DTx provides an incremental benefit over existing therapies. A good example would be the digital therapy RESET-O®, developed by Pear Therapeutics. This DTx approach has been developed for the treatment of opioid use disorder and has been cleared by the US Food and Drug Administration (USFDA). The clinical trial that led to the approval of RESET-O® is a randomized controlled trial in which the efficacy of DTx has been compared to standard buprenorphine therapy, a drug commonly used for treating opioid use disorder. This trial demonstrated that the addition of DTx to best pharmacologic therapy can significantly enhance clinical outcomes in patients with a history of abuse of opioids (11).

### Active control

In this case, the control arm is subjected to a further form of digital intervention in the form of digital therapy. This allows for a comparison of the efficacy of two distinct digital therapies. A case in point is the randomized controlled clinical trial conducted on the two digital therapies SLEEPIO® and SHUTi®, both of which are used for the treatment of insomnia (12).

### No treatment

The participants forming the control group do not receive any form of treatment. A good example is the randomized controlled trial of the digital therapy HAPPIFY®, which has been developed aiming at the reduction of states of anxiety and depression (13).

### Digital placebo (“sham”)

Control group patients receive a digital placebo. FDA defines a sham as an ineffective device, simulated procedure, or possibly a drug or biological product, which is used under conditions designed to resemble as closely as possible the conditions of use under investigation. In pharmacological studies, sham is considered to be the equivalent of placebo control. Designing a digital placebo is a complicated process. In the case of Software as Medical Devices (SaMDs), where treatment relies solely on software, the creation of an equivalent placebo control presents a significant challenge, which even the FDA acknowledges as “*it can be challenging to construct a placebo control that appears to function similarly to the device being tested but provides no therapeutic benefit*”. In randomized clinical trials for DTx, a digital active placebo is often used:a device that is identical to the study drug but does not deliver the therapeutic intervention (14). This helps minimize variability in results caused by the patient's user experience. A good example is a randomized controlled trial of the digital therapy Deprexis®, developed to treat depression. In this study, the Sham is an application providing generic wellness content (15). Another example is the clinical study designed for the validation of DTx Somryst® by Pear Therapeutics. In this trial, a distinct software program was utilized as a control to assess the efficacy of the treatment for insomnia. This control software was designed to include only minor elements of the software to be tested. The biggest challenge in designing a digital placebo is the removal of a digital therapy delivery component without compromising the patient's user experience. It is important that during the design phase of the DTx, no component not identified as a ‘digital active substance’ are removed, since those have not been attributed to active therapeutic properties.

Given the extensive scope of DTx and its diverse applications, it is reasonable to conclude that having a universal approach to the design of control conditions is unlikely to be effective. Some authors have proposed that, given the general low-risk profile of DTx, a minimal control or even a wait-list comparison could be an appropriate option in many cases. Although these may overestimate treatment effects, they may be a valid option for studying large population in a real-world context.

After examining the state of the art of clinical trials conducted in Italy on digital therapeutics, another crucial aspect concerns the regulatory dimension, which defines the regulatory framework applicable to DTx.

The aim is to provide stakeholders interested in commercializing a DTx with an overview of the European regulatory landscape and to compare it with the current Italian framework, which is still under development, in order to highlight the strengths and potential criticalities of regulatory approaches across Europe. At present, the regulatory situation in Italy remains fragmented, representing a potential obstacle for stakeholders seeking to commercialize a DTx. In particular, from an access-to-therapy perspective, Italy currently lacks a specific regulatory framework establishing reimbursement pathways and clear criteria for the identification and classification of digital therapeutics within the broader context of medical devices. For this reason, we conducted a comparative analysis with three European countries (Germany, France, and Belgium) which are currently more advanced in these areas, with the aim of identifying the respective advantages and disadvantages of each national approach.

For regulatory purposes a DTx, being a software application, is classified as medical device.

In the European Union (EU), the first law to regulate this type of device was the European Directive 93/42/EEC (16), adopted on 14 June 1993 and recognized in Italy with Legislative Decree 46/97 (17). This regulation has made it possible to establish a harmonized regulatory framework necessary for the design, manufacture and marketing of medical devices.

Article 1 of this directive contains the definition of medical device. It is defined as “*any instrument, apparatus, plant, substance or other product used alone or in combination, including software intended by the manufacturer to be used specifically for diagnostic and/or therapeutic purposes and necessary for its proper functioning, and intended by the manufacturer to be used in humans for the purpose of:*

- Diagnosis, prevention, control, treatment or alleviation of a disease
- Diagnosis, control, therapy, alleviation or compensation of an injury or handicap
- Study, replacement or modification of anatomy or a physiological process
- Intervention on conception

*whose principal action in or on the human body is not achieved by pharmacological or immunological means or by metabolism, but whose function can be assisted by these means"* (16).

Looking at this definition, it is possible to understand how the term *software* is used in the European Directive 93/42/EEC only to indicate a part of the medical device that is important for the operation of the device itself —-. The concept of “*stand-alone software*” — which can itself constitute the medical device, is therefore not taken into consideration.

In addition to providing a first definition of medical device, the European Directive 93/42/EEC introduced a classification system for medical devices, based on the risk associated with their use. Within the Directive, devices are divided into four main classes:

1. Class I (low risk)
2. Class IIa (medium risk)
3. Class IIb (medium-high risk)
4. Class III (high risk)

To identify the most appropriate class to associate with each medical device, it is important to consider two factors, namely the intended use and the intrinsic risk associated with the use itself.

This classification system plays a crucial role in the marketing of medical devices. It outlines the specific regulatory requirements that must be met to obtain the CE mark, which is mandatory for marketing devices within the EU. Depending on the class to which the medical device belongs, a simple self-certification by the manufacturer (for low-risk devices) may be required for the purpose of entry into the European market, rather than a review by an independent notified body (for high-risk devices).

In addition to providing a definition and classification of medical devices, the European Directive 93/42/EEC is important because of the strong emphasis placed on the safety and efficacy of the devices themselves. To obtain the CE mark, manufacturers of this type of device must demonstrate not only that their product is safe in its use for both the patient and healthcare professionals, but also that the device brings a benefit to the patient, and this benefit is significant. To ensure this, appropriate clinical evaluations are required, which provides scientific evidence for the efficacy and safety of the device and its intended use (18).

A final crucial aspect of European Directive 93/42/EEC is the introduction of post-market surveillance medical devices. According to this Directive, manufacturers are required to monitor their devices throughout the life cycle of the product, from placing in the European market until the end of marketing, and to report any incident or safety issues to the competent authorities. This is crucial to ensure any timely action in the event of incidents or issues, which further underlines the European Directive's key focus on safety while using these devices (19).

In 2007, following the increase in development of software technologies, the European Community issued the new Directive 2007/47/EEC (20) which broadens the definition of medical device and states that it can also be represented by SaMD. Directive 2007/47/EEC, recital 6 specifies that “*A software is itself a medical device when it is specifically intended by the manufacturer to be used for one or more of the medical purposes set out in the definition of a medical device. Even when used in a healthcare context, generic software is not a medical device.”* Additionally, Directive 2007/47/EEC, recital 20 states that “*Given the growing importance of software in the medical device industry, either as stand-alone or as embedded software in a device, a key requirement should be the validation of software according to the state of the art*”.

This new Directive 2007/47/EEC describes how software can be identified as a device itself and is no longer seen just as a component suitable for its operation. In 2017, EU approved a new European Regulation 2017/745/EC on medical devices, known as the Medical Device Regulation (MDR) (21). This new Regulation 2017/745/EC was published in the Official Journal of EU on 05 May 2017, with the aim of replacing the previous European Directive 2007/47/EEC (was replaced on 26 May 2020; date was postponed by one year due to the emergency of COVID-19 pandemic). On 05 August 2022, Legislative Decree No. 137 was published in the Official Gazette of the Italian Republic, which mandated all the necessary provisions to adapt Italian national legislation in place of European Regulation 2017/745/EC.

Compared to the previous Directive 2007/47/EEC, Regulation 2017/745/EC has some substantial differences. Since it is a Regulation, it is a legislative act that need not be transposed by the Member States of the EU but must be applied and respected in all its parts.

Also, this Regulation modified the earlier definition of medical device, inserting in it the functions of prognosis and prediction of a pathology.

To define software as a medical device, the Recital 19 in the Regulation 2017/745/EC is of fundamental importance. It states that “*It should be clarified that software intended by the manufacturer to be used for one or more of the medical uses indicated in the definition of medical device is considered to be a medical device, while software intended for general purposes, even if used in a healthcare context, or software for purposes associated with lifestyle and well-being is not a medical device. The qualification of software, either as a device or as an accessory, is independent of the location of the software or the type of interconnection between the software and a device”.*

The Regulation 2017/745/EC, therefore, emphasizes the indication of use as a discriminating factor to establish whether or not the software under consideration can be considered a medical device. According to the Recital 19, it is not sufficient for the software to track or record lifestyle data in order to fall within the definition of medical device, but it is necessary that such data can be used for the purposes of prevention, diagnosis, monitoring, treatment or alleviation of a disease.

The European Medical Device Regulation 2017/745/EC provides a classification based on the risk for medical devices and classified them into four classes as detailed below:

- Class I: Includes devices that present less criticality. Most of them are non-active and non-invasive.
- Class IIa: Includes devices of medium criticality, both non-active (invasive and non-invasive) and active, which interact with the body in a non-dangerous way.
- Class IIb: Includes medium/high criticality devices, both non-active (mostly invasive) and active, which could mine the safety of the patient
- Class III: Includes medium/high criticality devices, mostly implantable, which contain drugs.

This classification is determined by the intended purpose and potential risk of harm of the device, affecting the degree of regulatory oversight and conformity assessment needed. Higher risk classes require more stringent conformity assessment by a notified body.

Despite the introduction of a new classification, in the European Regulation there is no specific treatment related to DTx, which allows them to be classified in an unambiguous and adequate way. In Annex VIII, under Rule 11, it is stated that “*software intended to provide information used to make decisions for diagnostic or therapeutic purposes falls within class IIa, unless such decisions have such effects as to cause:*

- *Death or irreversible deterioration in the state of health of a person, in which case it falls within class III, or*
- *A severe deterioration in a person's health condition or surgery, in which case it falls into class IIb.*

*Software intended to monitor physiological processes is classified as class IIa, unless it is intended to monitor vital physiological parameters, where the nature of the variations in those parameters is such that, it is likely to create an immediate hazard to the patient, in which case it is classified as class IIb. All other software falls into class I”.*

Therefore, while the Regulation makes it explicit that software can be used to provide data or to monitor physiological processes, there is no point that refers to their use as a therapy. Since there is no therapeutic use for software, any medical device that has such a purpose should be classified as class I device under the category “*all other software”,* for the purposes of the Regulation.

Medical devices belonging to the class I are the devices for which the European Regulation does not provide for the intervention of an independent notified body for the purposes of CE certification. In this scenario, self-certification of the device by the manufacturer is sufficient. The Regulation therefore risks classifying heterogeneous software medical devices in any of the classes, which can have *devices* characterized by a moderate or high level of risk for the patient. For these, more structured safety assessments would be required. Hence, in the European market, this is one of the main reasons for many Digital Therapies to be part of Class I.

Additionally, there are two other reasons for DTx to be placed in class I. First is that the devices are defined as “*legacy”,* i.e., devices covered by a certification obtained before the entry of the Regulation 2017/745/EC and valid according to the transition rules set out in Article 120 of the Regulation itself. Next are the devices that have been erroneously attributed to class I by the manufacturer. It is important to highlight that classifying a medical device as class II or higher, and involving a notified body, has a considerable impact on the procedure while placing the product in the market, both in terms of the time required for the evaluation process, and to have an outcome of the evaluation (not necessarily positive), and, finally, the cost for obtaining and maintaining the certification. Therefore, the key problem to be resolved is the right classification of the medical device.

After this Regulation, two more documents were published by the Medical Device Coordination Group, established by Article 103 of the same Regulation, which provide further details regarding the classification of software as medical devices.

The first document entitled “MDCG 2019-11 - *Guidance on Qualification and Classification of Software in Regulation (EU) 2017/45 – MDR and Regulation (EU) 2017/46 – IVDR*”, was published in October 2019 (22).

This document specifies that “*Software intended to process, analyze, create, or modify medical information may qualify as medical device software if the creation or modification of such information is governed by a specific medical purpose*.” This means that software used in the medical field but for non-medical purposes is not classified as a medical device.

The guideline contains a more comprehensive classification method in the Annex III, which states that “*Usability of the* International Medical Devices Regulators Forum *(IMDRF) risk classification framework in the context of the MDR*”. Table 2 compares the patient's clinical status (Non-serious, Serious, Critical) with the impact on diagnosis or treatment of the patient, with an aim to interpret Rule 11 of Annex VIII of the MDR.

**Table 2:** Software as medical device categories (22).

State of Healthcare situation or condition	Significance of information provided by SaMD to healthcare decision		
	Treat or diagnose	Drive clinical management	Inform clinical management
Critical	IV	III	II
Serious	III	II	I
Non-serious	II	I	I

The IMDRF classification framework “ *SaMD: Possible Framework for Risk Categorization and Corresponding Considerations”*, was published in October 2014 by the IMDRF. This framework based the classification of a SaMD on:

- The impact of the information provided by the software on the healthcare professional decision;
- The patient health condition.

In the guideline, it is evident how software that drives of inform the clinical management about non-serious conditions are classified as class I (23).

It is also worth noting the use of the term “*to treat”* in Table 3*,* to underline the possibility of treating a pathology with a SaMD. In fact, “therapeutic purposes” is not a defined criteria in the EU MDR or in MDCG 2019-11 SaMD and, thus, it could be argued that intended purposes like prediction and prognosis, newly introduced in the definition of a medical device under the EU MDR, do not correspond to “therapeutic purposes”. Thus, leading a SaMD with such purposes to correspond to “other software” under Rule 11 and falling into Class I, as stated into Regulation 2017/745/EC Annex VIII. The IMDRF unofficial classification method would make it possible to categorize DTx in a more appropriate way, fully recognizing their added value.

**Table 3:** MDCG 2019-11 classification guidance on rule 11.

**State of Healthcare** **situation or patient condition**	**Significance of Information provided by the MDSW to a healthcare situation related to diagnosis/therapy**		
	**High** Treat or diagnose – IMDRF 5.1.1	**Medium** Drive clinical management – IMDRF 5.1.2	**Low** Informs clinical management (everything else)
**Critical** situation or patient condition∼IMDRF 5.2.1	**Class III** Category IV.i	**Class IIb** Category III.i	**Class IIa** Category II.i
**Serious** situation or patient condition∼IMDRF 5.2.2	**Class IIb** Category III. ii	**Class IIa** Category II.ii	**Class IIa** Category I.ii
**Non-serious** situation or patient condition (everything else)	**Class IIa** Category II.iii	**Class IIa** Category I.iii	**Class IIa** Category I.i

Reinterpreting the IMDRF classification framework, Annex III of the MDCG 2019-11 directly excludes Class I SaMD and according to the classification methodology described in the MDCG 2019-11 Annex III table below, a SaMD cannot fall into Class I, but is categorized, based on the level of risk associated with its use, into Class IIa, IIb or III only. MDCG 2019-11 reports a single example of Class I SaMD i.e., a mobile app intended to support conception by calculating the user's fertility status by tracking and predicting ovulation, based on a validated statistical algorithm.

The second document is “*MDCG 2021-24 - Guidance on Classification of medical devices*”, which was published by MDCG in October 2021. The aim of this document is to provide essential information to the manufacturers and provide guidance on the risk class when this needs to be determined. In addition, the document is a guideline for the classification as stated under the Annex VIII of the European Regulation 2017/745/EC (24), providing examples helping the understanding of the manufacturers during their classification process.

The Table 4 provides a tangible example to assist manufactures in the classification of their medical devices and interpreting the classification rules of the MD Regulation.

**Table 4:** MDCG 2021- 24 risk class examples for SaMD (24).

Class	Rule 11	Examples
IIa	Software intended to provide information which is used to take decisions with diagnosis or therapeutic purposes is classified as class IIa, except if such decisions have an impact that may cause:	• MDSW intended to rank therapeutic suggestions for a health care professional based on patient history, imaging test results, and patient characteristics, for example, MDSW that lists and ranks all available chemotherapy options for BRCA-positive individuals. • Cognitive therapy MDSW where a specialist determines the necessary cognitive therapy based on the outcome provided by the MDSW.
III	— death or an irreversible deterioration of a person's state of health (1), in which case it is in class III; or	• MDSW intended to perform diagnosis by means of image analysis for making treatment decisions in patients with acute stroke.
IIb	— a serious deterioration of a person's state of health or a surgical intervention, in which case it is classified as class IIb.	• A mobile app intended to analyse a user's heartbeat, detect abnormalities and inform a physician accordingly. MDSW intended for diagnosing depression based on a score resulting from inputted data on patient symptoms (e.g., anxiety, sleep patterns, stress etc.).
IIa	Software intended to monitor physiological processes is classified as class IIa,	• MDSW intended to monitor physiological processes that are not considered to be vital. • Devices intended to be used to obtain readings of vital physiological signals in routine check-ups including monitoring at home.
IIb	except if it is intended for monitoring of vital physiological parameters, where the nature of variations of those parameters is such that it could result in immediate danger to the patient, in which case it is classified as class IIb.	• Medical devices including MDSW intended to be used for continuous surveillance of vital physiological processes in anaesthesia, intensive care or emergency care.
I	All other software is classified as class I.	• MDSW app intended to support conception by calculating the user's fertility status based on a validated statistical algorithm. The user inputs health data including basal body temperature (BBT) and menstruation days to track and predict ovulation. The fertility status of the current day is reflected by one of three indicator lights: red (fertile), green (infertile) or yellow (learning phase/cycle fluctuation).

Given the growing importance of DTx for patients and healthcare systems, it is considered necessary to contribute to the discussion on their introduction in Italy, identifying their benefits on a solid scientific basis, removing obstacles to their diffusion, promoting their accessibility through funding at the national level. Following the Covid-19 emergency, Italy has seen a notable surge in interest regarding the adoption of digital and innovative therapeutic solutions. However, this momentum is dampened by the lack of a legislative framework that regulates their clinical validation. Consequently, the topic of the benefits and opportunities associated with reimbursement mechanisms is more distant, despite them being increasingly evident in the global scenario.

#### DTx in Europe and across Italy

In Europe, use of DTx is limited. The integration of DTx for patient access and care pathways are available in few countries only. Although DTx is governed by EU Medical Device Regulations, there is a lack of harmonisation in the regulatory requirements due to differences in interpretation. The three main challenges highlighted by the European Federation of Pharmaceutical Industries and Associations (EFPIA) are:

- Challenges in the harmonisation of evidence requirements and lack of value assessment processes
- No standardised or specific reimbursement pathway for DTx in most countries

- There is inadequate funding, and DTx reimbursement pathways do not ensure uptake (25)

Currently, regulations are in place in Germany, France and Belgium, and DTx policy pathways are evolving across Europe (26). Although each state has different laws and regulations, there are some fundamental factors, which are common to all member states of the EU:

1. **Data security and protection** - The European reference legislation is the General Data Protection Regulation (GDPR), as well as compliance with the standards defined by the ISO 27001 certification.
2. **Clinical evidence -** A certification that certifies the software as a medical device (SaMD), is always required and as well as an appropriate clinical evaluation is needed, which analyses the usability and clinical benefits of the device (27).

In order to assess strengths and weaknesses inherent in each national system, we performed an analysis involving Germany, France, and Belgium regulatory pathways. Each of these countries has, at least partially, integrated digital therapeutics into its reimbursement system and therefore offers valuable insights for the potential future development of Italian legislation in this area.

#### Germany

Germany was the first state in EU to introduce a regulation in the legislation regarding the use of digital therapies. On 7 November 2019, German Parliament approved the Digital Healthcare Act (Digitale-Versorgung-Gesetz, DVG), following which digital applications for health care (Digitale Gesundheits Anwendungen, DiGA) were integrated into the German healthcare system and made reimbursable by the latter (28). The DiGA are medical devices classified as class I or class IIa and used either by the patient or by the patient and the doctor (29).

DiGA are monitored in all their certification phases by the German Federal Institute for Medicines and Medical Devices (BfArM). This institute carries out a preliminary evaluation of the digital therapy, approximately for three months, following which the institute provides a provisional or permanent approval, or non-approval of the digital therapy (30).

In the case of provisional approval, the BfArM temporarily enters the DiGA in the register of reimbursable therapies. Within twelve months of entry of DiGA in the register, the manufacturer must submit sufficient evidence to demonstrate the health benefits of the device for the patient, to remain in the register. The provisional approval is provided only once. At the end of the twelve months, if the final approval is refused, manufacturer has to wait for one year before submitting a new application (30).

When DiGA receives permanent approval, it is directly approved as a reimbursable therapy, as all the requirements are met. After entry in the register, the manufacturer can set the price of the therapy, which will be valid for the first year of marketing. Subsequently, the price will be agreed upon by a comparison between the manufacturer of the DiGA and the German Health Insurance Association and will be valid from the thirteenth month after entry into the market (31).

If the final price of the therapy is lower than the price established by the manufacturer, then the manufacturer must return the difference in value to the state. Therefore, this is a potential and critical point, as cases have been reported, where the manufacturers after a year of marketing the DiGA, had to return substantial amount of money to the state. This is true in cases where, the prices decided in discussion with the Association were found to be much lower than the price established by the manufacturers (30).

A strong point in support of the German system is the fact that digital therapies are reimbursable, even if the therapies are only provisionally approved. The fact that immediate reimbursement is available, is helpful for the manufacturers, as they can invest or divert considerable resources into the conduct of necessary studies for further data collection, which may aid in demonstrating the real clinical benefit of their products.

#### France

On 30 March 2023, with decree no. 2023-232, the French Ministry of Health and Prevention authorised the coverage of medical expenses by health insurance, for a maximum of twelve months, for all medical devices falling within the category of potentially innovative digital technologies through a program named “Prise En Charge Anticipée Numérique, PECAN” (32).

The French program aims to provide a fast-track reimbursement process for digital health applications and medical devices.

Category includes medical devices designed for the purpose of providing therapy, which are included in the list of reimbursable digital services and products (Liste des Produits et Prestations Remboursables, LPPR) and medical devices designed to ensure monitoring activity, which are instead part of the medical monitoring activities (Liste des Actes et des Prestations Médicales, LATM) (33). The procedure requires companies to submit a dossier to HAS which is responsible for Health Technology Assessment following the granting of a CE marking.

For the purposes of evaluation by the French “Medical Device and Health Technology Evaluation Committee (CNEDIMTS)”, the manufacturer submits a complete dossier regarding the medical device. The CNEDIMTS is responsible for evaluating the CE marking of the medical device reported by the manufacturer, as well as the clinical benefit, added value provided by the product presente (34) and appraisal of these dossiers.

The economic assessment (when required) is performed by the Economic and Public Health Evaluation Committee (CEESP - Commission d'évaluation économique et de santé publique). The CNEDiMTS recommendation is then submitted to the Economic Committee of healthcare products (CEPS) for the pricing negotiation. The final decision for registration of the reimbursement list falls under the jurisdiction of the Ministers of Health and of Social Security and is published in the Official Journal.

Similar to Germany, the PECAN procedure (35) allows for fast-track evaluation with early reimbursement by health insurers for one year, even without conclusive clinical evidence of the application's effectiveness. During the first year, manufacturers receive a monthly predefined compensation fee per patient. After one year, if the solution shows clinical benefits and is approved by the authorities to be a PECAN, the price can be negotiated for DTx but remain fixed for telemonitoring solutions.

To request this early access, it is necessary to submit a simultaneous application to:

1. The Digital Health Agency (ANS) to obtain the solution's certificate of conformity with the DMN interoperability and security framework;
2. The High Authority for Health (HAS) and the Ministers of Health and Social Security to assess its innovative nature, particularly in terms of clinical benefit or progress in the organization of care.

PECAN process aims to complete evaluation and validation process in 90 days [60 days for the assessment by the Digital Health Agency (ANS) and the National Authority for Health (HAS) + 30 days for the final decision by the French Ministry of Health]. Upon receipt of favorable opinions, the decision on early access is published by decree of the Ministers of Health and Social Security within 30 days (36). Once the early coverage is established, there are 6 months to apply for the registration of the devices in the LPPR (List of Reimbursable Products and Services).

The manufacturer must provide all the data required for a definitive evaluation of the device. The French government also pays close attention to the type of economic impact that the adoption of new digital therapies can have on the healthcare system: in particular, DTx manufacturers can now present, within the dossier, a budget impact analysis that illustrates the advantages brought by the digital therapy in terms of healthcare expenditure. The data that can be obtained from an analysis of this type are very useful in order to define the appropriate price for the therapy presented, which takes into account the clinical benefit brought, the costs and the potential savings for the healthcare system.

Consequently, France opened its market to German digital medical devices in risk classes IIb and III, even though Germany's own regulatory framework was limited to class IIa devices at that time (37). However, in the last year, by means of the “Digital Act Digital-Gesetz”, (DigiG) law (37), which come into force on 26 March 2024, there was an extension of the potential prescribability at the expense of the GKV as DiGA to medical devices of risk class IIb (38).

Nevertheless, France remains an interesting market in two ways: firstly, as a market for exporting DiGA that is already listed in Germany, and secondly, as a market for digital medical devices that are not certified in Germany because of their risk class.

#### Belgium

Mobile Health Belgium represents the national reference platform for CE-marked mobile medical applications, centralizing all relevant and essential information on digital health solutions that comply with the criteria defined by the federal government.

The aim of the platform is to guide citizens, healthcare professionals and healthcare institutions through the huge number of apps (applications) that exist within the healthcare system, for all types of purposes (diagnostics, therapy, monitoring, prognosis, prediction, prevention and more) and in all types of diseases or therapeutic fields. It is structured as a three-level validation pyramid. Introducing a ‘Fast-Track’ for DTx evaluation, assessing technical and evidence prerequisites (39).

An application always enters the lowest level M1 and can move up the hierarchy via level M2 to the highest level M3.

Basically, to receive the green light, it is necessary to reach the top of the validation pyramid for mHealth technologies and pass the FAMHP (Federal Agency for Medicines and Health Products) assessment. Here below the main steps:

Level M1: Assessment of CE marking and GDPR compliance.
Level M2: Assessment of data security risks, medical data privacy, connectivity and interoperability.
Level M3: Clinical and socio-economic demonstration of the added value brought by the solution.

The overall management of mHealthBelgium (40) is carried out by beMedTech (industry federation of the medical technology industry) and Agoria (association representing companies active in the technology sector), in close cooperation with 3 national authorities: Federal Agency for Medicine and Health Products (FAMHP, the competent authority responsible for the safety, quality and efficacy of medicines and health products, responsible for level M1); eHealth Platform (federal eHealth organization building the infrastructure for information exchange in the health sector, responsible for level M2); NIHDI (National Institute for Health Insurance and Disability, responsible for the reimbursement of health products and services, level M3).

Level 3 plus is reached when the socio-economic value of the medical device is demonstrated and become eligible for reimbursement definitively funded by the NIHDI, the National Institute for Health Insurance and Disability. This financing can be temporary or permanent. The temporary reimbursement is for applications of an innovative nature, for which there is already evidence, but some uncertainties remain. This will be shown as M3 light = M3-. When the use of mobile medical applications is definitively integrated into the financing of the care process, it will be indicated as M3+ (41).

For three years, DTx in Belgium can be reimbursed by the National Institute for Health and Disability Insurance. The duration of the provisional authorization is usually three years. During this time, the health application is already reimbursed by the health insurance companies. The price is negotiated by the applicant with the NIHDI. During the provisional reimbursement phase, the applicant must report to the NIHDI after 18 months on the progress of the implementation plan for final approval. The complete documentation for the decision on permanent approval and reimbursement must be submitted no later than 6 months before the end of the trial period (41).

#### Italy next steps

Despite increasing political attention toward digital therapeutics, Italy currently remains in a transitional regulatory phase. The Italian regulatory landscape is still characterized by fragmentation, which may hinder stakeholders intending to bring a DTx to market.

Specifically, with regard to patient access, there is no dedicated national framework defining reimbursement mechanisms or establishing clear criteria for the recognition and classification of digital therapeutics within the medical device regulatory system. The proposal of an Italian model of accessibility for digital therapies can take advantage of the examples already available in other countries, despite the widespread (but rapidly evolving) lack of funding, not only for DTx but also for most Digital Health and Digital Medicine solutions (42). At present, the Italian system remains in a pre-implementation stage.

On 07 June 2023, the Honorable Simona Loizzo, President of the Digital Health and Digital Therapies Parliamentary Intergroup, presented to the Chamber of Deputies the proposed law n.1208, entitled “Provisions on digital therapies” (43). This is the first legislative proposal in Italy that aims to regulate this emerging sector of medicine.

The text of the proposal is composed of 4 articles:

- Art. 1 - Definition of digital therapies
- Art. 2 - Evaluation Committee of digital therapies
- Art. 3 - Permanent observatory on digital therapies
- Art. 4 - Inclusion in the LEA

From the structure of the text, it is clear that this is a proposed delegation law, through which to establish a framework of principles and criteria to be followed to regulate the specific matter of digital therapies in procedural terms.

Discussion is ongoing between the various parliamentary bodies and the proposed law could be subject to amendments, additions, and other proposals for changes.

On 16 October 2024 and most recently on 30 January 2025, two additional legislative proposals were presented: law proposal no. 2095 (44) by Quartini and others, and law proposal no. 2220 by Girelli (45) and others, respectively. Both proposals, entitled “Provisions on digital therapies,” incorporate some of the basic recommendations of the Loizzo law, although with some differences.

The above legislative proposals are examples of the political effort that Italy is carrying on with the aim to regulate digital therapies as has already happened in other countries, firstly attributing them a role within the National Health Service.

Starting first and second quarter of 2025, the Commission is conducting an investigation on the law proposals and has concluded a series of hearings to gather input and views from industry experts and major health institutions. The next phase involves passing of the proposals to a dedicated committee so that a basic text of the measure can be adopted and then proceed with the amendment phase and final votes. The ideal scenario is to have a final approval of the text by the end of 2025.

In addition, every new health technology, including digital therapies, must be subjected to HTA (Health Technology Assessment). This assessment today becomes necessary to determine the therapeutic value and position in the care pathway of Digital Technologies for Health, with the aim of standardizing decisions relating to purchase, reimbursement and use (46).

The concept of health technology is broad and includes Health technologies encompass medicinal products, medical devices, *in vitro* diagnostic medical devices and medical procedures, as well as measures for disease prevention, diagnosis or treatment (47). These technologies therefore consist of all the practical applications of knowledge, including digital therapies, which are used to promote health and prevent, diagnose and treat diseases.

It is worth highlighting that Italy is also adapting its regulatory framework to the HTA Regulation and everything related to technological innovation. In the past, tools such as the Pact for Health 2014-2016 and Law 190/2014 (48) had laid the foundations for HTA in our country, which were then consolidated, with regard to medical devices, by Italian legislative decrees 137 and 138 of 2022.

In line with the provisions of the HTA Regulation and with the vision of the National Recovery and Resilience Plan (PNRR), the Ministry of Health adopted the National HTA Program for medical devices [PNHTA 2023-2025 (49)] in 2023, with the aim of:

- Integrating HTA into decision-making processes at national, regional and corporate levels;
- Using HTA throughout the entire life cycle of technologies, from design to obsolescence;
- Improving efficiency in the allocation of healthcare resources.

For the above purpose, the National HTA Program 2023-2025, includes a series of integrations including all the aspects that impact the governance of technologies in the NHS:

- National and Regional Telemedicine: HTA on medical devices for Telemonitoring (PNRR Mission 6 Component 1 subinvestment 1.2.3) (50);
- Development of digital solutions to improve access to and quality of healthcare services: Specific evaluation, authorization and reimbursement framework for DTx and artificial intelligence AI (51).

In addition, AGENAS “Agenzia nazionale dei servizi sanitari regionali” which has assumed the role of National Agency for Digital Health, as per Legislative Decree 27 January 2022 n. 4 (52), converted with amendments by Law 28 March 2022 n. 25 (53), has started a new partnership with Farmindustria “Association of pharmaceutical companies” aimed at developing and implementing innovative projects in the field of healthcare digitalization, including telemedicine. The collaboration also aims to improve the research and training sector, thanks to the development of a project that aims to:

- Create an HTA evaluation system and a specific framework for approval and reimbursement for DTx, in order to facilitate patient access to effective therapies;
- Establish criteria for the analysis of healthcare data resulting from the use of artificial intelligence (AI) systems, including guidelines, outcome evaluation metrics and procedures for sharing data among all actors involved in the sector, both public and private.

The initiative is in line with the European Health Data Space (EHDS) Regulation (54), in order to:

- Ensure individuals have greater access to their digital health data, both nationally and across borders, allowing them to have greater control over this information and promoting a single market for medical devices and AI systems;
- Create an integrated and efficient system for the real-time collection of health data (RWD) and for the analysis of this information for research (RWE), improving the effectiveness of therapies and stimulating innovation in the sector;
- Define regulations that encourage the secondary use of health data.

The 2025 Budget Law provides for the updating of the PNHTA by the Ministry of Health by 30 June 2025 (55).

In the pharmaceutical sector, the Italian Medicines Agency (AIFA) plays a primary role. The Scientific and Economic Commission of Medicine (CSE), established in 2024, provides opinions on clinical evaluation parameters, in order to ensure alignment between national policies and the European regulatory framework.

In the past months, on 19th February 2025 AIFA President, Robert Giovanni Nisticò, spoke at the hearing at the Social Affairs Commission of the Chamber of Deputies as part of the fact-finding activity phase on the draft laws regarding DTx.

The President audited by the Commission expresses his positive thoughts as follows: “*Digital therapies can represent an opportunity to strengthen public health by integrating traditional therapies with new tools for the management of diseases to be used exclusively or in combination with drugs or to enhance the effectiveness of the treatment with undoubted advantages for the patient and for all the subjects who, directly and indirectly, intervene in the therapeutic process, including the Italian Medicines Agency. Aifa is available to provide support and to issue any competent scientific opinions relating to the effectiveness and safety of digital therapies*” (56).

In conclusion, the proposed laws aim to fill the regulatory gap that exists in Italy by regulating digital therapies and establishing their role within the national health service. The strengthening of the regulatory framework can enable the adoption and reimbursement of digital therapies, ensuring high quality standards and equitable access for patients. We hope that the regulation of digital therapies represents the future of healthcare in Italy so that, following the example of other European countries, chronic diseases can be prevented, managed, and treated through software that generates positive clinical outcomes.

Unlike the other analyzed countries, where structured access pathways have already been implemented, Italy has not yet translated political intent into an executable regulatory model. The current debate reflects institutional engagement but does not yet provide manufacturers with predictable timelines, eligibility criteria, or procedural clarity.

#### Clinical interest of a DTx

Digital therapies represent a significant evolution in the treatment of various diseases due to their ability to integrate advanced technology-based therapeutic interventions, remote monitoring and personalised management of clinical conditions. These tools, which are supported by robust scientific evidence, offer the potential to optimise chronic disease management, improve treatment adherence and monitor therapeutic response in real time. This has significant implications for both improved clinical outcomes and healthcare system efficiency. From the perspective of the pharmaceutical industry, DTxs represent a twofold opportunity. Firstly, they serve to expand the available therapeutic arsenal, and secondly, they enable companies to consolidate their position within the care cycle by developing integrated solutions that promote personalised and data-driven care.

According to global research conducted by McKinsey & Company, a majority of patients are willing to use digital health services, provided they meet the required standard of care. This data indicates a shift in patients’ preferences, who are looking for digital solutions to complement conventional treatments. The growing adoption of platforms such as Apple Health Kits, which facilitate the monitoring of vital parameters and health-related habits, and research kits that enable the development of digital therapeutic solutions, underscores the advancement towards a more personalised medicine approach. This phenomenon presents significant strategic opportunities for pharmaceutical companies, particularly through collaborative efforts with technology companies and academic institutions.

DTxs offer a key clinical benefit by enabling the collection of real-time data on patients’ health status and improving the monitoring of physiological parameters, allowing treatments to be adjusted in a timely manner and optimising the management of chronic diseases, reducing the risk of complications and hospital admissions. The integration of DTx with technologies such as artificial intelligence and wearable devices further enhances the predictive capacity of treatment models, making diagnosis and treatment processes more efficient. The collection of real-world data (RWD) through these technologies also enriches scientific evidence and supports new drug development by integrating clinical and behavioural data.

The expansion of the DTx market, which has the potential to equal or even exceed the growth of traditional medicines, is likely to stimulate further investment in the sector, thereby exerting a direct impact on therapeutic innovation. The integration of DTx into traditional healthcare systems, facilitated by evolving regulatory frameworks, will encourage the adoption of more integrated, personalised and data-driven care models. This will result in the establishment of more efficient healthcare management systems that will be capable of responding in a timely and accurate manner to patients’ clinical needs, thereby improving the quality of care and overall therapeutic effectiveness.

In summary, investment in DTx represents a strategic opportunity for the pharmaceutical industry. These therapies offer an innovative response to emerging clinical needs and enable the consolidation of a leading position in the global healthcare landscape, fostering increasingly personalised and sustainable medicine.

From a strategic perspective for stakeholders interested in commercializing a digital therapeutic, it is also necessary to consider additional factors, including Manufacturers’ post-marketing obligations and some considerations if the manufacturer is also the MAH.

Under the Regulation (EU) 2017/745 on medical devices (MDR), manufacturers of medical devices placed on the Italian market are required to fulfill several post-marketing obligations to ensure ongoing safety and performance of their products.

These obligations are part of a broader European framework that aims to protect public health and ensure that devices continue to meet the required safety standards once they are available for use. Manufacturers are required to establish and maintain an effective Post-Market Surveillance (PMS) system.

This system should collect and assess data from clinical use, monitor the safety and performance of the device, and identify potential risks. It helps manufacturers respond promptly to safety issues or adverse events that may arise during the device's life cycle (Article 83, MDR) (57).

One of the key elements of post-marketing obligations is the vigilance system. If any serious incident or near miss occurs, manufacturers must report it to the Italian Ministry of Health and the relevant authorities within strict timelines (Article 87, MDR) (58).

A serious incident is defined as an event that might lead to the death or serious deterioration in the health of a patient or user. If a risk is identified, the manufacturer must take appropriate corrective actions (e.g., device recall, modification, or update of instructions for use).

These actions must be communicated to the relevant stakeholders, including users, healthcare professionals, and regulatory authorities (Article 89, MDR) (57). For high-risk devices, manufacturers are required to submit Periodic Safety Update Reports (PSUR).

These reports summarize the safety profile and performance of the device, based on data collected through PMS activities. The reports must be submitted at regular intervals to the regulatory authorities, including the Italian Ministry of Health, and demonstrate continuous compliance with safety standards (Article 86, MDR). Manufacturers are also required to register their devices in the European Database on Medical Devices (EUDAMED), which centralizes information on all devices placed on the EU market. This includes submitting information on the device's safety and performance, as well as reporting any incidents (Article 31, MDR).

The more updated definition regarding vigilance on medical device are defined into European guideline MDCG 2023-3 “Questions and Answers on vigilance terms and concepts as outlined in the Regulation (EU) 2017/745 on medical devices” (58).

The issue of the prescription status of digital therapy remains unresolved. This responsibility is expected to remain with the attending physician, who must choose the most suitable tool based on its quality, effectiveness, and the patient's therapeutic needs—particularly important given that the tool must be funded by the NHS. Similarly, unlike other healthcare systems where the dispensing process is well-defined, the proposal makes no mention of a distribution channel. In the case of apps, one would expect this to be represented by a platform connected to healthcare systems, from which patients could then download the prescribed treatment.

Finally, the issue of advertising and promotion for these devices which confines them within article 26 of Legislative Decree 137/2022 which expressly prohibits advertising to the public for medical devices subject to medical prescription, a restrictive prohibition for this category of tools that require active patient participation and HCPs direction.

Another important aspect of DTx is that they allow to collect a massive quantity of post-marketing patient-level data that can be harnessed to re-assess their safety and effectiveness in real-world setting. However, the increase in individual patient-related data poses concerns about data privacy and quality, thus highlighting the need to define a legal framework that allows on the one hand to guarantee individual privacy and on the other hand to transparently share data for research purposes (59).

From the point of view of a Pharmaceutical Company, which is Manufacturer or Distributor of a DTx, on one side there are the requirements related to the medical device and from the other side also the collection and the management of any pharmacovigilance information must be guaranteed. It is important to put in place an adequate system which consent to maintain the overview not only on the device but on the information reported trough the device.

It is important to define the correct management and exchange of patient-related data, if the Pharmaceutical Company have a contract with a Manufacturer/Distributor for a DTx is important to define and cover the management and the exchange of all safety information, including both materiovigilance and pharmacovigilance responsibilities.

A vigilance clause must be included into the Service Agreement and a Vigilance Agreement which covers both materiovigilance and pharmacovigilance responsibilities must be stipulated between the Parties.

#### Limitations of the review

The primary contribution of this review is to provide a structured overview of the current Italian landscape from the perspective of a company intending to commercialize a DTx. Specifically, we aim to identify the existing regulatory and reimbursement constraints, the emerging institutional opportunities, and the potential future developments that may shape market access in Italy. By contextualizing the Italian framework within the broader European environment, and particularly in comparison with countries that have already implemented more advanced regulatory and reimbursement pathways, we seek to offer stakeholders a practical interpretative framework to support strategic decision-making.

At the same time, this analysis presents certain limitations. The comparative regulatory assessment focuses exclusively on European Union countries. On the one hand, this choice allows for a more meaningful and coherent comparison, given the shared supranational regulatory context and similarities with the Italian healthcare system. On the other hand, it excludes consideration of regulatory and reimbursement models developed in non-EU countries, such as the United States, where digital therapeutics have evolved within a different institutional and market structure. Moreover, the clinical trials analysis relies exclusively on registrations in ClinicalTrials.gov; trials registered in other databases (e.g., EU Clinical Trials Register) or published after this date are not captured.

This review primarily reflects the perspective of the authors, who are employees of the same pharmaceutical company, which may introduce an industry perspective into the assessment of regulatory and reimbursement frameworks, particularly regarding market access considerations; readers should interpret the findings with this potential bias in mind. While this work does not incorporate the viewpoint of regulatory authorities, it is the authors’ position that providing this perspective, which remains partially underexplored in the current literature, is nonetheless of importance. Finally, the perspective of regulatory authorities and payers has not been incorporated, which represents a further potential source of imbalance in the analysis.

## Discussion and conclusion

Digital therapies offer a set of key benefits that are transforming healthcare. The capacity of these digital therapies to monitor patients in real time facilitates improved adherence to prescribed treatments and enables clinicians to identify early warning signs, thereby preventing deterioration and enabling the tailoring of treatments. The potential for accessing DTx via mobile devices enhances their accessibility and discretion, particularly in sensitive domains such as mental health. This improves treatment efficacy and reduces waiting times.

Digital therapies permit a high degree of personalisation of treatments, enabling the adaptation of therapies to the specific needs of each patient and thus improving clinical outcomes. Furthermore, they provide a cost-effective alternative in settings with limited healthcare resources, thereby reducing overall costs and facilitating treatment in remote locations. In particular, DTx are effective in the management of chronic diseases, supporting patients in self-management through educational tools and continuous monitoring, enabling clinicians to observe vital signs and symptoms even at a distance.

Access to DTx is restricted by infrastructural limitations, such as the need for a reliable internet connection, as well as by challenges like the insufficient training of healthcare professionals—particularly in rural or economically disadvantaged communities. Management of data security is also a significant challenge. The utilisation of DTx entails the processing of sensitive patient data, necessitating the implementation of advanced protection systems and the attainment of clear informed consent, which is frequently complex and not readily comprehensible to patients themselves. It is therefore imperative that the central tenets of privacy and patient autonomy are upheld, as is the question of liability in the case of therapeutic decisions made by algorithms.

A further challenge is the need for greater explainability and trust in DTx. The decision-making processes that are frequently based on algorithms can be opaque and challenging to comprehend for both patients and physicians. This can impede the transparent evaluation of risks and benefits. Additionally, there is a potential for unintentional discrimination due to inherent biases in the algorithm training data, which could have a detrimental impact on vulnerable patient populations. It is imperative to elucidate the legal liability associated with clinical outcomes attained through the utilisation of DTx, particularly in instances where the influence of artificial intelligence is predominant.

The digital divide represents a significant risk of inequity, as inequalities in access to the internet and technology may result in the creation of new barriers to health. Furthermore, health systems must address the issue of financial burden, as digital technologies are not always included in reimbursement plans, which could result in the diversion of resources from other health sectors. Furthermore, the promotion of DTxs by public institutions is constrained by legislation, necessitating an examination of the optimal distribution channel to facilitate their adoption without contravening restrictive regulations.

In conclusion, digital technologies in healthcare represent a significant advance, with the potential to enhance accessibility, efficiency and quality of care. However, the adoption of these technologies necessitates a balanced approach that addresses ethical and technological concerns, including issues of fairness, privacy, and accountability. It is only through the proactive addressing of these issues that the integration of DTx into healthcare systems can be achieved in a responsible manner, ensuring effective use that respects patients’ rights.
